# A Morphometric Analysis of the Middle Cranial Fossa in Mexican Adults for Dolenc and Kawase Approaches With Computed Tomography, 3D Reconstruction and Dry Skulls

**DOI:** 10.7759/cureus.99476

**Published:** 2025-12-17

**Authors:** Edgar Nathal, Alejandro Serrano Rubio, Alejandro Becerril-Mejía, Ambar Elizabeth Riley-Moguel, Manuel Angeles-Castellanos, Karen Eloisa Xochipa-Ruiz, José Carlos Rocha-Villegas, Zahira Elizabeth Medina-Félix, Dora Yvette Lugo-Hilario

**Affiliations:** 1 Vascular Neurosurgery, National Institute of Neurology and Neurosurgery "Manuel Velasco Suárez", Mexico City, MEX; 2 Anatomical Sciences, Faculty of Medicine, Universidad Nacional Autonoma de Mexico, Mexico City, MEX; 3 Neurosurgery, Hospital Juárez de México, Mexico City, MEX; 4 Neurosurgery, Instituto de Seguridad y Servicios Sociales de los Trabajadores del Estado (ISSSTE) Tláhuac, Mexico City, MEX

**Keywords:** computed tomography (ct), dolenc approach, dry skull, kawase approach, mexican adults, middle cranial fossa, skull base morphometry

## Abstract

Background: Accurate middle cranial fossa (MCF) morphometry is crucial for the safe application of middle fossa approaches such as the Dolenc and Kawase approaches, which expand exposure to the cavernous sinus and petroclival region. These parameters vary widely among individuals and populations, making population-specific data important. We therefore collected MCF measurements in Mexican adults using both CT scans and dry skulls to establish normative values and assess the accuracy of CT scans.

Methods: Two cohorts (97/107 subjects each) were analyzed: head CT scans (in vivo) and dry skulls (ex vivo) of adult Mexicans. Key distances (Foramen ovale (FO) - Foramen spinosum (FS), FO - arcuate eminence (AE), FS - AE) and innominate pillar thickness were measured bilaterally (each in duplicate). CT versus skull differences were tested (α = 0.05) using side-to-side symmetry analysis, and the 5th/95th percentiles were used as anatomical limits.

Results: CT and osteology showed close concordance for FO-FS, supporting CT-based extradural orientation for the middle fossa approach. In contrast, FO-AE and FS-AE were larger on dry skulls by a few millimeters, indicating that routine CT slightly underestimates the drillable petroclival window relevant to Kawase. The innominate pillar was consistently present and appeared slightly thicker on CT (partial-volume effect), remaining a practical landmark to localize CN V3 (mandibular division of the trigeminal nerve) between FO and FS. Left-right asymmetry was not evident on CT; osteology showed only small AE-related leftward tendencies. Percentile guardrails (P5, P50, P95) provide pragmatic bounds for preoperative planning.

Conclusions: This study provides the first MCF morphometric norms for Mexican adults to support safer, individualized Dolenc, Kawase, and middle fossa approach planning. CT-based measurements are largely reliable for surgical planning, although they slightly underestimate the Kawase approach reference points.

## Introduction

The middle cranial fossa (MCF) and cavernous sinus are distinct skull-base compartments with different neurovascular contents. The MCF houses the petrous segment of the internal carotid artery (ICA), the Gasserian (trigeminal) ganglion, and branches of cranial nerve V (trigeminal), branches V2 and V3. It includes key foramina: the* foramen ovale* (FO) transmits the mandibular nerve (V3) and an accessory meningeal artery, and the *foramen spinosum* (FS) transmits the middle meningeal artery, middle meningeal vein, and the nervus spinosus (meningeal branch of V3). The arcuate eminence (AE) is a bony prominence on the petrous temporal bone indicating the underlying superior semicircular canal [1-3].

In contrast, the cavernous sinus is a paired dural venous plexus adjacent to the sella turcica that contains the cavernous ICA and abducens nerve (CN VI) within its lumen, with cranial nerves III, IV, V1, and V2 running in its lateral wall [1,3]. Both regions have traditionally been considered surgically challenging due to their densely packed neurovascular contents. Modern extradural-intradural techniques have expanded safe exposure in this region. The Dolenc (frontotemporal extradural) approach includes an extradural anterior clinoidectomy, allowing early exposure of the clinoid and ophthalmic ICA segments while reducing temporal lobe retraction [1-4]. The Kawase (anterior petrosectomy) approach involves removing a triangular petrous apex window bounded by the arcuate eminence, greater superficial petrosal nerve, petrous ICA, and trigeminal nerve; because the apex lacks critical neural tissue, this drilling is relatively safe and offers excellent access to the prepontine cistern and upper clivus [1-4]. In practice, the Dolenc and Kawase approaches are used for cavernous or carotid artery aneurysms, mid-basilar aneurysms, petroclival meningiomas, trigeminal schwannomas, and selected clival or ventral brainstem lesions. They can also be combined (Dolenc-Kawase) to create a broad corridor from the anterior cavernous sinus to the interpeduncular cistern for large or complex petroclival tumors [2,5].

Anatomical studies show considerable variability among individuals in MCF landmarks; FO size and shape distributions have been reported in dry skull collections of different populations, showing cohort-dependent ranges and accessory patterns [1,5]. FS calibre, morphology, and prevalence also differ in European (Croatian/Slovenian) and South Indian series. In addition, imaging-based work has characterized petrous roof thickness and the AE-superior semicircular canal relationship in Chinese and Japanese populations [1,6]. Notably, most prior reports focus on a single structure (FO or FS) rather than corridor-level relationships; few relate foramina to the AE or to approach-specific drilling windows. Such variability, along with possible population effects, highlights the importance of population-specific morphometry to plan safe drilling corridors [7,8].

Accordingly, the objective of this study is to characterize morphometric variation of the MCF in a Mexican adult population, providing national reference data to guide safe surgical planning and reduce the risk of neurovascular injury during skull-base procedures.

## Materials and methods

Study design and setting

An observational, descriptive morphometric study was conducted at tertiary academic hospitals and an institutional osteological collection in Mexico City. The objective was to quantify MCF bony relationships relevant to the middle fossa approach and Dolenc and Kawase (anterior petrosectomy) corridors in Mexican adults and to provide clinically useful reference values (means, ranges, and percentiles).

Study population and specimens

Two independent cohorts were analyzed.

CT Cohort (In Vivo)

Ninety-seven adult Mexican patients (age ≥20 years) who underwent head CT scans and had no otologic or skull-base pathology on imaging were included. This cohort comprised approximately 50 male and 47 female subjects, with a mean age of 45 years (range ~20-80 years). Exclusion criteria for this group were any history of skull-base surgery or significant head trauma, mass lesions or dysplasia affecting the MCF region, motion or metallic artifacts precluding measurement, or incomplete demographic/side data on the scan.

Dry Skull Cohort (Ex Vivo)

We examined 107 adult Mexican crania (dry skulls) in good preservation from an institutional osteology collection (Universidad Nacional Autónoma de México, UNAM). The collection included both male and female skulls (approximately 65 male and 42 female, based on available records), all estimated to be adults (skeletal maturity roughly corresponding to age ≥20 years at death). Exclusion criteria for skull specimens included any fractures or deformity involving the sphenoid or petrous region, post-mortem damage to relevant foramina or eminences, or uncertain laterality of the specimen.

Both cohorts were bilateral by design; sides with absent/indistinct landmarks were excluded for that variable only.

Imaging acquisition (CT)

Non-contrast multidetector CT of the head acquired with 0.5-0.6 mm slice thickness (sub-millimetric, near-isotropic voxels) reconstructed with a bone kernel. Scans were reviewed and measured in a DICOM Viewer, using standardized bone window settings and consistent zoom. Multiplanar reconstructions (MPR) were generated in axial, coronal, and sagittal planes, complemented by oblique views optimized for the petrous apex: the Stenvers plane (oblique along the long axis of the petrous ridge) and the Pöschl plane (orthogonal to the Stenvers plane, aligned with the plane of the superior semicircular canal). These obliques reduce partial-volume effects, allowing for the reliable identification of the arcuate eminence (AE) and adjacent petrous surfaces.

**Figure 1:**
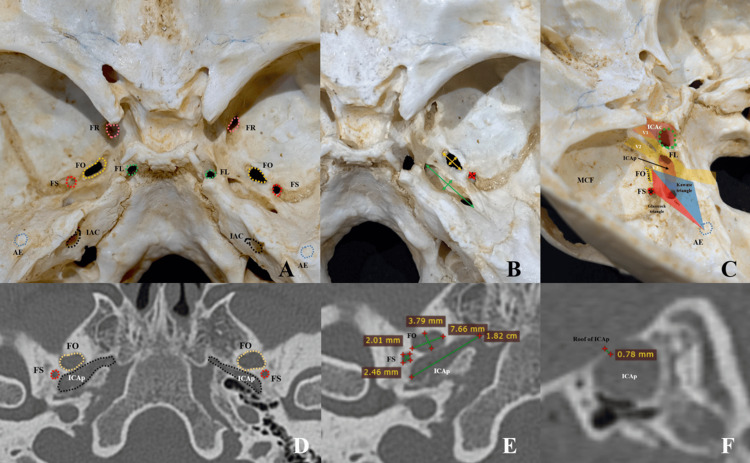
Middle cranial fossa landmarks for Glasscock and Kawase triangles (rhomboid area): osteology and CT workflow. (A) Endocranial view of the middle cranial fossa showing the foramen ovale (FO) and foramen spinosum (FS) bilaterally, the foramen rotundum (FR) and foramen lacerum (FL), and the bony strut between FO and FS (innominate pillar, IP). Dashed outlines highlight foramina. (B) Oblique osteologic view emphasizing the spatial relationship of FO/FS with the arcuate eminence (AE) on the petrous ridge; the IP is interposed between FO and FS. (C) Left middle cranial fossa view demonstrating the anatomical limits of the Kawase triangle (blue) and the Glasscock triangle (red). The internal carotid artery petrous segment (ICAp) is outlined, as well as the trajectory of the trigeminal nerve divisions (V1 and V2). The arcuate eminence (AE, blue dotted circle) serves as a posterior landmark. These triangles delineate key surgical corridors to the petrous apex and adjacent skull base structures. (D) Coronal CT (bone window) identifying FO and FS and the petrous internal carotid artery canal (ICAp); these slices were used to confirm foraminal centers and safety margins. (E) Stenvers reconstruction showing the measurement workflow on the oblique plane: interforaminal FO–FS, minimum thickness of the innominate pillar (orthogonal to FO–FS; red calipers), and FO–AE/FS–AE distances (green calipers). Numeric overlays are illustrative of caliper output. (F) Pöschl reconstruction demonstrating assessment of thin bony partitions along the Kawase trajectory; example shows the roof thickness over the ICAp (~0.78 mm in this case). Abbreviations: AE, arcuate eminence; FO, foramen ovale; FS, foramen spinosum; FR, foramen rotundum; FL, foramen lacerum; ICAp, petrous internal carotid artery canal; PL, innominate pillar; IAC, internal auditory canal.

In total, 194 petrous regions were assessed (97 left, 97 right). Landmarks were identified on the best-fit plane for each structure and cross-checked in orthogonal planes. Linear distances were obtained with the on-screen caliper, recorded in millimeters, and defined as follows: 1) FO-FS: center-to-center distance between the FO and FS. 2) FO-AE / FS-AE: shortest straight-line distance from the center of FO or FS to the apex of the AE on the oblique plane where the AE was most prominent. 3) Innominate pillar (IP) thickness: minimum bone thickness of the strut between FO and FS, measured orthogonally to the FO-FS line on the slice showing the narrowest waist (Figures 1,5).

All measurements were obtained under blinded conditions. For CT, studies were de-identified and reviewed in randomized order; for dry skulls, catalogue labels were masked during measurement. The primary observer (neurosurgeon) measured all variables bilaterally in duplicate on two separate sessions, and the mean of the duplicates was analyzed. To assess reproducibility, a second independent observer (e.g., neuroradiologist/anatomist) re-measured a random subset of 97 CT petrous sides and 107 dry-skull sides, also in duplicate and blinded to the primary readings. Inter-observer agreement was summarized with intraclass correlation coefficients (ICC, two-way random, absolute agreement; single measures) for each variable and side; intra-observer repeatability was estimated from duplicate reads. Outlying values (>3 SD from cohort mean) triggered re-inspection in orthogonal planes (CT) or caliper repositioning (skulls); if a landmark was indistinct, that side was excluded for that variable only.

Osteological protocol (dry skulls)

Linear dimensions were obtained with digital calipers (Mitutoyo Corp; Kanagawa, Japan; resolution 0.01 mm); angular measures were obtained with a goniometer. Each variable was recorded bilaterally in duplicate and averaged. Skulls with focal erosion or post-mortem defects affecting the specific landmark were excluded from that measurement.

Anatomical landmarks and variable definitions

Measurements were taken in millimeters (mm) unless otherwise specified; angles in degrees (°), side is reported as Right/Left: 1) FO and FS diameters: maximal (long axis) and minimal (short axis) apertures on the measurement plane. 2) FO-FS distance (Dolenc corridor): the linear center-to-center distance between FO and FS on the axial plane that best depicts both foramina (Figures 1,5). On skulls, the geometric center was approximated by bisecting the long and short axes of each aperture. 3) FO-AE and FS-AE (Glasscock corridor): linear distance from the FO/FS geometric center to the apex of the AE on the petrous surface. AE was identified as the bony ridge overlying the superior semicircular canal; when subtle, the most prominent arcuate crest was chosen, confirmed in orthogonal planes. 4) IP thickness: minimal cortical thickness of the bony strut between FO and FS measured orthogonally to the FO-FS line at its narrowest segment. 5) Parasellar angle: The angle formed by the FO-FS vector relative to the anteroposterior skull-base axis on the axial plane, as per institutional protocol.

Measurement protocol and quality control

All measurements were performed with the CT workstation electronic caliper and the same digital calipers for skulls. Bilateral, duplicate measurements were recorded and averaged. Values deviating by more than 3 SD from the cohort mean were re-checked against the source images/skull; obvious data entry errors were corrected, and outliers were retained to preserve anatomical variability. The measurement order (Right→Left) was alternated across cases to minimize systematic bias.

Outcomes

Primary readouts were FO-FS, FO-AE, FS-AE, and IP thickness (bilaterally). Secondary readouts included foraminal diameters (FO/FS long and short axes) and the parasellar angle. The clinical intent was to generate guardrails for extradural Dolenc orientation and for the extent of anterior petrosectomy in Kawase.

Sample size 

This was a descriptive atlas; no a priori power calculation was performed. A census-style sampling strategy was used to maximize precision and reduce selection bias: all consecutive CT examinations meeting inclusion criteria within the study window and all available, well-preserved dry skulls in the institutional collection were included. The final sample comprised 97 CT heads (194 petrous sides) and 107 dry skulls; side-specific Ns for each variable reflect occasional exclusion when a landmark was indistinct for that measurement only. To document the achieved precision, we computed 95% confidence-interval half-widths using the observed dispersions. 

Statistical analysis

Continuous variables are reported as mean ± SD and range (min-max). For clinically actionable lookup, percentiles (P5, P50, P95) were generated for key distances. No hypothesis testing was planned. Analyses were performed in Python (v3.13.0; Python Software Foundation, Wilmington, DE, USA), using *pandas *for data handling and *matplotlib *for figure generation.

Software and reproducibility

Python (v3.13.0; Python Software Foundation, Wilmington, DE, USA), *pandas*, and *matplotlib *were used. Scripts to reproduce descriptive tables/figures are available on reasonable request, together with de-identified measurement tables.

## Results

A total of 97 skull CT scans and 107 dry skull specimens were analyzed for the core measurements. The sample size varied slightly by measurement: in the CT cohort, both right and left sides were available for all 97 cases for distances such as FO-FS, FO-AE, FS-AE, pillar thickness, and the parasellar angle. In the dry skull cohort, measurements were obtained from 107 right/107 left sides for FO-FS and pillar thickness, from 105 right/104 left sides for FO-AE, and from 105 right/104 left sides for FS-AE. Any side in which a landmark could not be clearly identified was excluded from the analysis of that specific variable (while still being included for other measurements where landmarks were distinct). An overview of the middle cranial fossa landmarks and their spatial relationships relevant to the Dolenc and Kawase approaches is illustrated in Figure 1.

Glasscock corridor

**Figure 2:**
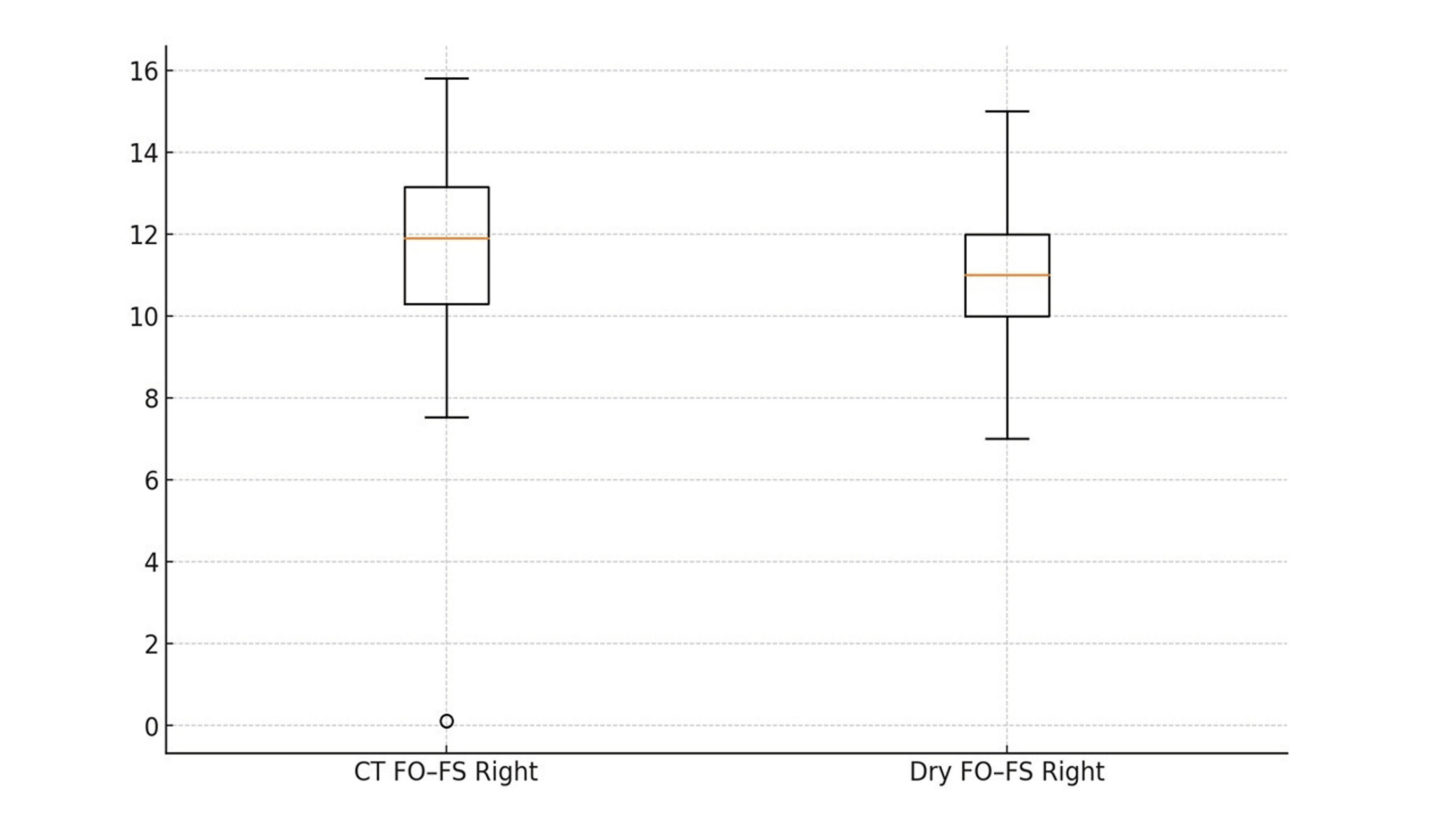
FO-FS (Right): CT vs Dry skulls (boxplot, units: mm) Inter-foraminal distance from foramen ovale (FO) to foramen spinosum (FS) on the right side in CT and adult dry skulls. CT on orthogonal multiplanar reconstructions; direct calipers on skulls (0.01 mm). Boxplots show median, IQR, whiskers to 1.5×IQR; outliers as points. CT–skull concordance supports CT-based planning of Dolenc’s extradural corridor (lateral cavernous sinus/Meckel’s cave).

On CT, the distance between the FO and FS averaged 11.74 ± 2.20 mm on the right side and 11.80 ± 2.02 mm on the left side. The distribution of FO-FS distances in the CT scans was relatively broad (right side P5 ≈ 8.72 mm, median 11.90 mm, P95 14.72 mm; left side P5 9.23 mm, median 11.78 mm, P95 14.96 mm), with ranges of 0.11-15.80 mm (right) and 1.45-16.70 mm (left). In dry skulls, the FO-FS distance was similar, measuring 11.06 ± 1.48 mm on the right and 11.25 ± 1.59 mm on the left (right side P5 9.00 mm, median 11.00 mm, P95 14.00 mm; left side P5 9.00 mm, median 11.00 mm, P95 13.35 mm). The ranges observed in dry specimens were 7.00-15.00 mm (right) and 3.00-14.50 mm (left). The CT measurements closely matched the osteological measurements for FO-FS on both sides (Figure 2), which supported the use of CT morphometry to plan the extradural trajectory in a Dolenc approach (peeling of the lateral cavernous wall and opening Meckel’s cave).

FO-AE Distance

For the distance from the foramen ovale to the arcuate eminence (FO-AE), the CT scans showed a mean of 24.22 ± 4.16 mm on the right side and 23.80 ± 3.59 mm on the left side. The FO-AE distance on CT had a right-side range of 2.56-32.00 mm (with P5 18.66 mm, P50 24.20 mm, P95 29.97 mm) and a left-side range of 15.30-32.50 mm (P5 17.62 mm, P50 24.20 mm, P95 29.22 mm). In the dry skulls, the FO-AE distances were larger: 30.19 ± 2.69 mm on the right and 31.70 ± 2.16 mm on the left. The dry skull FO-AE ranges were 22.00-36.00 mm (right) and 27.00-38.00 mm (left), with right-side P5 26.00 mm, median 30.00 mm, P95 34.00 mm, and left-side P5 28.00 mm, median 32.00 mm, and P95 35.00 mm.

FS-AE Distance

For the distance from the foramen spinosum to the arcuate eminence (FS-AE), CT measurements averaged 20.13 ± 3.74 mm on the right and 20.09 ± 3.41 mm on the left. The CT ranges were 2.12-29.80 mm (right) and 13.90-31.20 mm (left), with right-side P5 14.86 mm, median 20.20 mm, P95 25.36 mm, and left-side P5 14.98 mm, median 19.90 mm, and P95 25.02 mm. In dry skulls, the FS-AE distance was again higher: 22.77 ± 3.04 mm on the right and 23.72 ± 3.08 mm on the left. Dry skull ranges were 16.00-30.00 mm (right) and 18.00-33.00 mm (left), with right P5 18.00 mm, median 23.00 mm, P95 28.00 mm, and left P5 19.00 mm, median 23.50 mm, P95 29.00 mm.

**Figure 3:**
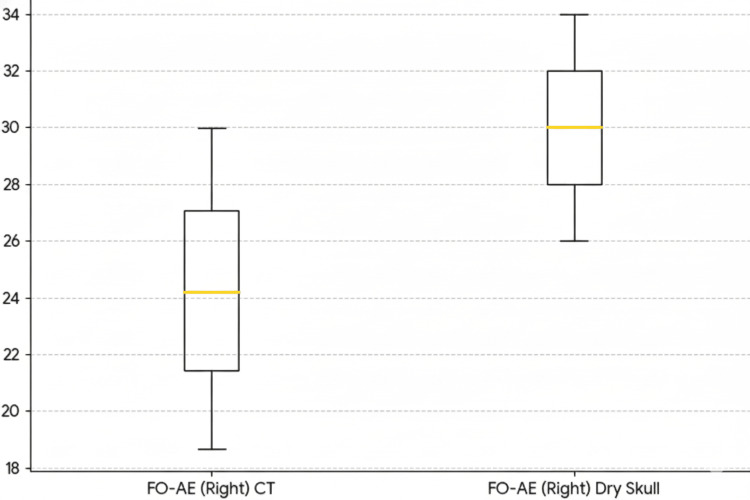
FO–AE (Right): CT vs Dry skulls (boxplot, units: mm) Boxplots show the foramen spinosum (FO)–arcuate eminence (AE) distance for CT and dry skulls on the right and left sides (CT: n=97/97; dry skulls: n=105/104). Boxes represent the interquartile range (Q1–Q3) with the median line; whiskers indicate the 5th–95th percentiles; points denote outliers. In both sides, FO–AE is shifted upward in dry skulls relative to CT, illustrating the systematic underestimation of the petroclival drilling window by preoperative CT morphometry.

**Figure 4:**
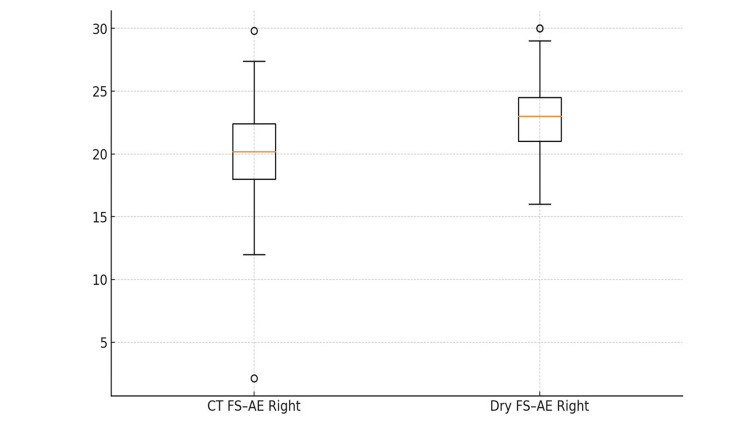
FS–AE (Right): CT vs Dry skulls (boxplot, units: mm) Distance from foramen spinosum (FS) to the arcuate eminence (AE) on the right side in CT and adult dry skulls. CT on orthogonal MPR; direct calipers on skulls (0.01 mm). Median, IQR, whiskers to 1.5×IQR; outliers as points. AE-related distances are larger on skulls.

**Figure 5:**
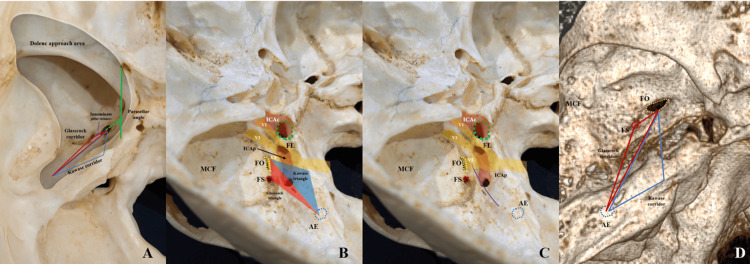
Middle fossa working corridors and measurement workflow (osteology and 3D reconstruction). (A) Superior view of the middle cranial fossa demonstrating the Dolenc approach area. The limits of the Glasscock corridor (red lines) and Kawase corridor (blue lines) are shown, together with the immediate paraclival and parasellar regions (green lines). These anatomical corridors delineate key surgical windows toward the parasellar and petrous apex regions. (B) Left middle cranial fossa view showing the Kawase triangle (blue), Glasscock triangle (red), and their relationship with FL, FO, and FS. The internal carotid artery petrous segment (ICAp) and intracavernous carotid (ICAc) are visualized, as well as the trigeminal nerve divisions (V1, V2). The arcuate eminence (AE, blue dotted circle) marks the posterior limit. (C) Left middle cranial fossa dissection highlighting the overlap between the Glasscock and Kawase triangles. The petroclival ICA (ICAp), FO, FS, and FL are shown as landmarks. The trigeminal nerve divisions (V1, V2) and AE define the anatomical boundaries relevant to the middle fossa approach. (D) Three-dimensional reconstruction illustrating the surgical Glasscock (red) and Kawase (blue) corridors from the middle cranial fossa. FO, FS, and AE are key reference points for identifying this approach. AE, arcuate eminence; FO, foramen ovale; FS, foramen spinosum; FL, foramen lacerum; ICAp, petrous internal carotid artery canal; MCF, middle cranial fossa; PL, innominate pillar; MMA, middle meningeal artery.

Notably, the distances from the foramina (FO and FS) to the arcuate eminence were consistently larger in the dry skulls than in the CT scans, by approximately 6-8 mm for FO-AE and 2-4 mm for FS-AE on each side (Figures 3, 4). In practical terms, this indicates that preoperative CT tended to underestimate the “petroclival bone window” available. Intraoperatively, surgeons often needed a slightly wider anterior petrosectomy than the CT measurements alone would suggest (while still staying within the safety limits of the greater superficial petrosal nerve and cochlea). The distribution and systematic differences in FO-AE distances between CT and dry skull measurements are visualized in Figure 5.

Innominate pillar thickness

The bony pillar between the FO and FS (the “innominate pillar”) was consistently identified in all specimens. On CT, the pillar’s thickness measured 2.56 ± 1.13 mm on the right and 2.36 ± 0.97 mm on the left (right range 0.64-5.98 mm, left range 0.72-4.58 mm; P5 1.07 mm, P50 2.43 mm, P95 4.49 mm on the right; P5 1.07 mm, P50 2.21 mm, P95 4.23 mm on the left). These CT values were slightly higher (by ~0.2-0.4 mm) than the dry skull measurements, which were 2.13 ± 0.96 mm on the right and 2.11 ± 1.13 mm on the left (right range 0.80-6.50 mm, left range 0.50-7.00 mm; P5 1.00 mm, P50 2.00 mm, P95 4.00 mm for both sides). This small systematic difference is likely due to a partial-volume averaging effect on CT. Importantly, the pillar was a reliable extradural landmark, useful for quickly identifying the location of the foramen spinosum (and the middle meningeal artery) and the foramen ovale (and the V3 trigeminal nerve) during Dolenc-type exposures.

Foraminal dimensions 

The size of key foramina varied between CT imaging and dry skulls but generally fell within expected ranges for skull-base anatomy. On CT, FO on the right side had a mean major axis of 7.24 ± 1.10 mm and a minor axis of 4.56 ± 1.09 mm (range 4.33-9.62 mm for the major axis and 2.66-7.66 mm for the minor). In the dry skulls, because of irregular shape, the FO was characterized by a single effective diameter measurement, averaging 6.26 ± 1.10 mm on the right and 6.47 ± 1.12 mm on the left (ranges 4.00-9.00 mm on both sides). For FS, CT measurements on the right showed a mean major axis of 2.16 ± 0.59 mm and a minor axis of 2.60 ± 0.68 mm (ranges 0.10-3.78 mm and 0.10-4.37 mm, respectively). On the left CT side, the FS major axis was 2.72 ± 0.78 mm and the minor axis was 2.09 ± 0.45 mm (ranges 1.33-7.76 mm and 1.02-3.84 mm). In the dry skulls, the FS diameter averaged 2.27 ± 0.65 mm on the right and 2.25 ± 0.68 mm on the left (ranges 1.00-5.00 mm and 1.00-3.50 mm, respectively). Overall, these foraminal dimensions were in line with those reported in prior skull-base studies, and they provide useful benchmarks for anticipating corridor widths during surgery. For instance, knowing these sizes helps in planning extradural peeling of the cavernous sinus region, guiding clip trajectories near the cavernous ICA, and facilitating catheter or nerve manipulation through the FO (see Appendices).

Parasellar angle

The parasellar angles (as measured by our institutional reference line) were also comparable between modalities. On CT, the parasellar angle averaged 40.13° ± 9.23° on the right and 39.34° ± 8.98° on the left (with dispersion from about the 5th to 95th percentile being 24.72° to 57.44° on the right, and 25.48° to 54.02° on the left). The dry skull measurements showed a slightly higher mean angle (approximately 50.12° ± 7.67° on the right and 51.17° ± 7.01° on the left), with 5th to 95th percentile ranges of 35.30° to 62.40° (right) and 40.00° to 61.00° (left). Despite the difference in absolute mean values between CT and dry skulls, both cohorts exhibited similar variability (standard deviation on the order of 7-9°) and no significant left-right asymmetry within each modality. Thus, for preoperative planning purposes, the CT-derived angles were considered clinically comparable to the angles measured in actual skulls. This supports using pre-op CT scans for angular orientation in the middle fossa approach and for identifying specific parasellar structures, as the CT angles closely reflected true anatomical orientation. Parasellar angle measurements by modality and side, including mean values and percentile distributions, are presented in Appendices.

Side-to-side symmetry and modality agreement

In general, measurements were symmetric between the right and left sides for key parameters. For example, the mean FO-FS distance and the pillar thickness were nearly identical on the right vs. left in both the CT and dry skull groups. Likewise, the systematic underestimation observed in CT for the AE-related distances (FO-AE and FS-AE) was bilateral and of similar magnitude on both sides.

Outliers and data variability

Values beyond the 95th percentile (extremely high values) occurred in the dataset for all variables and were retained by design to reflect true anatomical variability. These outliers were carefully checked; any obvious data-entry errors were corrected upon re-review, ensuring that the extremes represented real anatomical differences. For practical surgical planning, we found that referring to percentile values (P5, P50, P95) provided more meaningful “guardrails” than relying on absolute minima or maxima. For instance, although the smallest observed FO-AE distance on right-side CT was an extreme ~2.56 mm in one case, the 5th percentile for that measurement was around 18.66 mm. This suggests that using a lower-bound estimate like the P5 is more useful for anticipating anatomy than an isolated extreme value. In summary, the results were presented with their 5th, 50th, and 95th percentiles to give a robust sense of variability, which is more informative for planning than just the full range.

## Discussion

This study provides a descriptive atlas of middle cranial fossa (MCF) morphometry in Mexican adults, focused on landmarks that guide the middle fossa approach as well as the Dolenc and Kawase approaches. Three principal findings emerged: first, FO-FS distances showed close CT-CT osteology concordance bilaterally, supporting the reliability of CT-based planning for the extradural Dolenc middle fossa approaches; second, FO/FS-AE distances were consistently larger on dry skulls than on CT (≈6-8 mm for FO-AE; ≈2-4 mm for FS-AE), indicating that routine clinical CT tends to underestimate the petroclival window relevant to the identification of the petrous ICA and the anterior petrosectomy of Kawase and third; the innominate pillar was consistently present and only slightly thicker on CT (expected partial-volume effect), reinforcing its value as a practical landmark for localizing V3 between FS and FO.

Several technical factors likely explain the CT-skull discrepancy toward the arcuate eminence. Thin cortices and curved petrous surfaces are susceptible to partial-volume averaging and edge selection biases on sub-millimetric CT, particularly when the AE is low-profile [6,7]. In contrast, caliper measurements on bone capture the crest directly and may more faithfully follow the true bony relief. Methodological differences (center-to-center linear metrics on planar reconstructions vs. direct osteology) also contribute. Regardless, the systematic direction of the bias (CT underestimation) was bilateral and stable across variables, which is clinically actionable.

Implications for Dolenc

Because FO-FS is robust across modalities, CT morphometry can be trusted for extradural cavernous-sinus orientation, lateral wall peeling, and early ICA control. Percentile “guardrails” help frame planning: e.g., FO-FS Right (CT) P5≈8.7 mm, P50≈11.9 mm, P95≈14.7 mm; operating within these bounds reduces surprises when defining the working channel over Meckel’s cave [8-10].

Implications for Kawase

For the anterior petrosectomy, surgeons should anticipate that pre-op CT will slightly undercall the drillable apex. As a practical rule, plan for a modest increase in the petrosectomy, on the order suggested by our bias (a few millimeters), to obtain the intended ventral brainstem exposure, while rigorously respecting safety boundaries (GSPN trajectory, petrous ICA, and labyrinth). Here, percentile guardrails are useful bookends: e.g., FO-AE Right CT P95≈30.0 mm versus dry P5≈26 mm; FS-AE Right CT P95≈25.4 mm versus dry P5≈18 mm. Intraoperative navigation and microanatomical confirmation of the AE should arbitrate the final limits [11,12-16].

Role of the innominate pillar

The pillar’s constant presence and predictable thickness (CT mean ≈2.6 mm; dry ≈2.1 mm) make it a fast, reproducible extradural cue to index V3 between FO and FS during Dolenc-type exposures (Figures 1, 2). The slight CT thickening is expected; surgeons should interpret the structure qualitatively (orientation) rather than quantitatively (drilling depth) [17].

Strengths of this work include a dual-modality design (clinical CT and osteology), bilateral duplicate measurements, and reporting of clinically usable percentiles (P5-P95).

Limitations include unpaired cohorts, potential scanner/reconstruction heterogeneity, and definitional variability of the AE when low-relief angles were not harmonized across modalities, so they are presented descriptively only. We did not stratify by sex/age, nor did we analyze outcomes, and linear metrics on planar CT do not capture 3D surface curvature.

Future directions include paired CT-skull validation, micro-CT correlation to refine AE identification, automated 3D segmentation for surface distances, sex/age stratification in larger Mexican/Latin-American samples, and prospective linkage of morphometry to operative extent and morbidity.

## Conclusions

In Mexican adults, CT and osteology showed strong agreement for the FO-FS distance, supporting the use of CT morphometry to plan Dolenc and middle fossa approaches. In contrast, FO-AE and FS-AE distances were systematically larger on dry bone than on CT, indicating that routine CT slightly underestimates the Kawase petroclival window by a few millimeters. The innominate pillar proved to be a constant, practical landmark for rapid extradural localization of V3 and the FO after exposing the FS. Percentile-based guardrails derived from these data provide pragmatic bounds for preoperative planning and may help standardize safer skull-base drilling in the middle cranial fossa.

## Appendices

**Table 1:** CT morphometry (mm) Summary of bilateral linear measurements on non-contrast, high-resolution temporal-bone CT (0.5–0.6 mm; bone kernel). FO–FS: center-to-center distance between the foramen ovale (FO) and foramen spinosum (FS). FO–AE / FS–AE: shortest straight-line distance from the FO/FS center to the apex of the arcuate eminence (AE) on oblique Stenvers/Pöschl reconstructions. Pillar: minimum orthogonal thickness of the innominate pillar (bony strut between FO and FS). N = number of sides measured; Mean and SD are per side; Min–Max indicates the observed range. R/L = right/left. Measurements were performed bilaterally in duplicate; sides with indistinct landmarks were excluded for that variable only.

Metric	N	Mean	SD	Min	Max
FO-FS R	97	11.74	2.2	0.11	15.8
FO-FS L	97	11.8	2.02	1.45	16.7
FO-AE R	97	24.22	4.16	2.56	32
FO-AE L	97	23.8	3.59	15.3	32.5
FS-AE R	97	20.13	3.74	2.12	29.8
FS-AE L	97	20.09	3.41	13.9	31.2
Pillar R	97	2.56	1.13	0.64	5.98
Pillar L	97	2.36	0.97	0.72	4.58

**Table 2:** Dry skull morphometry (mm) Summary of bilateral linear measurements obtained on Mexican adult dry skulls using digital calipers (0.01 mm precision). FO–FS: centre-to-centre distance between foramen ovale (FO) and foramen spinosum (FS). FO–AE / FS–AE: shortest straight-line distance from FO/FS centres to the apex of the arcuate eminence (AE). Pillar: minimum orthogonal thickness of the innominate pillar (bony strut between FO and FS). N = number of sides measured; Mean and SD = per side; Min–Max = observed range. R/L = right/left. Damaged or indistinct sides were excluded for that metric only. This osteological dataset serves as a baseline reference for comparison with CT-based morphometry.

Metric	N	Mean	SD	Min	Max
FO-FS R	107	11.06	1.48	7	15
FO-FS L	107	11.25	1.59	3	14.5
FO-AE R	105	30.19	2.69	22	36
FO-AE L	104	31.7	2.16	27	38
FS-AE R	105	22.77	3.04	16	30
FS-AE L	104	23.72	3.08	18	33
Pillar R	107	2.13	0.96	0.8	6.5
Pillar L	107	2.11	1.13	0.5	7

**Table 3:** Percentile guardrails (P5, P50, P95; mm) Percentile reference values for side-specific measurements in Mexican adults from two cohorts: CT (0.5–0.6 mm, bone kernel) and dry skulls (digital calipers, 0.01 mm). P50 is the median; P5 and P95 provide conservative lower–upper guardrails to frame expected anatomic limits when planning Dolenc (FO–FS) and Kawase (FO/FS–AE) corridors. Pillar denotes the minimum orthogonal thickness of the bony strut between FO and FS. N = number of sides contributing to each percentile. Use modality-specific values (do not mix across modalities). Sides with indistinct or damaged landmarks were excluded for that metric only. FO, foramen ovale; FS, foramen spinosum; AE, arcuate eminence.

Variable	Modality/Side	N	P5	P50	P95
FO-FS	CT Right	97	8.72	11.9	14.72
FO-FS	CT Left	97	9.23	11.78	14.96
FO-AE	CT Right	97	18.66	24.2	29.97
FO-AE	CT Left	97	17.62	24.2	29.22
FS-AE	CT Right	97	14.86	20.2	25.36
FS-AE	CT Left	97	14.98	19.9	25.02
Pillar	CT Right	97	1.07	2.43	4.49
Pillar	CT Left	97	1.07	2.21	4.23
FO-FS	Dry Right	107	9	11	14
FO-FS	Dry Left	107	9	11	13.35
FO-AE	Dry Right	105	26	30	34
FO-AE	Dry Left	104	28	32	35
FS-AE	Dry Right	105	18	23	28
FS-AE	Dry Left	104	19	23.5	29
Pillar	Dry Right	107	1	2	4
Pillar	Dry Left	107	1	2	4

**Table 4:** Parasellar angle (degrees) by modality and side Parasellar angle measured with the institutional reference line. Values are reported as mean ± standard deviation and the 5th–95th percentiles (guardrails). Within each modality, right–left dispersion was similar; dry skull means were ~10° higher than CT, but variability (SD ~7–9°) was comparable.

Variable	Side	N	Mean ± SD (°)	P5 (°)	P95 (°)
Parasellar angle	CT Right	97	40.13 ± 9.23	24.72	57.44
	CT Left	97	39.34 ± 8.98	25.48	54.02
	Dry skulls Right	107	50.12 ± 7.67	35.30	62.40
	Dry Skulls Left	107	51.17 ± 7.01	40.00	61.00
